# Supplementary material for: Fast and sensitive GCaMP calcium indicators for imaging neural populations
Source: Nature. 2023 Mar 15;615(7954):884–91. doi: 10.1038/s41586-023-05828-9 (PMC10060165; doi:10.1038/s41586-023-05828-9)
Supplement: Supplementary file 1 — This file contains Supplementary Tables 3 and 4. Supplementary Table 3: Adult Drosophila L2 assay protocol. Supplementary Table 4. Summary of changes to the field stimulation pipeline between jGCaMP7 publication and current manuscript. See separate Excel files for Supplementary Tables 1 and 2. [file 41586_2023_5828_MOESM1_ESM.pdf]

nature portfolio

https://doi.org/10.1038/s41586-023-05828-9

Supplementary information

Fast and sensitive GCaMP calcium indicators for imaging neural populations

In the format provided by the authors and unedited

Supp. Table 3. Adult *Drosophila* L2 assay protocol

Cycle	Frequency	Off	On	Trials	Volt (Lights Off)	Volt (Lights On)	Time (s)	Description
1	0.5			10	0	5	20	Frequency
2		25	500	25	0	5	14	Dark Flash
3		8	500	25	0	5	12	Dark Flash
4		4	500	25	0	5	12	Dark Flash
5	0.5			10	0	5	20	Frequency
6	30			600	0	5	20	Frequency
7	28			560	0	5	20	Frequency
8	26			520	0	5	20	Frequency
9	24			480	0	5	20	Frequency
10	22			440	0	5	20	Frequency
11	20			400	0	5	20	Frequency
12	18			360	0	5	20	Frequency
13	16			320	0	5	20	Frequency
14	14			280	0	5	20	Frequency
15	12			240	0	5	20	Frequency
16	10			200	0	5	20	Frequency
17	8			160	0	5	20	Frequency
18	6			120	0	5	20	Frequency
19	4			80	0	5	20	Frequency
20	2			40	0	5	20	Frequency
21	1			20	0	5	20	Frequency
22	0.5			10	0	5	20	Frequency
23		2000	500	15	Ramp	5	45	Ramp
24		1000	500	15	Ramp	5	23	Ramp
25		3000	500	15	Ramp	5	67.5	Ramp
26		25	500	25	2.5	5	12	Dark Flash
27		25	500	25	0	2.5	12	Dark Flash
28		8	500	25	2.5	5	14	Dark Flash
29		8	500	25	0	2.5	14	Dark Flash
30		500	25	25	0	5	14	Light Flash
31		500	8	25	0	5	12	Light Flash
32		500	4	25	0	5	12	Light Flash
33	0.5			10	0	5	20	Frequency

Supp. Table 4. Summary of changes to the field stimulation pipeline between jGCaMP7 publication[1] and current manuscript:

	jGCaMP7 publication[1]	Current publication	Reason for change
**Cell segmentation algorithm**	Script with manually set thresholds	Cell segmentation with Ilastik[2], a machine learning-based algorithm	Better separation of somata from neuropil
**Source of neurons**	Neonatal rat hippocampus	Neonatal rat cortex	More neurons can be harvested from the cortex, enabling higher throughput
**Imaging speed**	35 Hz	200 Hz	Focus on kinetics in current publication
**Quality control**	Wells with low expression manually removed from analysis	Wells with low expression manually removed from analysis; dim outlier cells automatically excluded	Dim cells (F_0 near background) produce artificially high $\Delta F/F_0$ signal

1. Dana, H. *et al.* High-performance calcium sensors for imaging activity in neuronal populations and microcompartments. *Nat Methods* **16**, 649–657 (2019).
2. Berg, S. *et al.* ilastik: interactive machine learning for (bio)image analysis. *Nat Methods* **16**, 1226–1232 (2019).
