# Supplementary material for: Fast and sensitive GCaMP calcium indicators for imaging neural populations
Source: Nature. 2023 Mar 15;615(7954):884–91. doi: 10.1038/s41586-023-05828-9 (PMC10060165; doi:10.1038/s41586-023-05828-9)
Supplement: Supplementary file 2 — Reporting Summary [file 41586_2023_5828_MOESM2_ESM.pdf]

nature portfolio

Corresponding author(s): Loren Looger, Karel Svoboda, Jeremy Hasseman, Sam Wang, Glenn Turner

Last updated by author(s): 12/19/2022

Reporting Summary

Nature Portfolio wishes to improve the reproducibility of the work that we publish. This form provides structure for consistency and transparency in reporting. For further information on Nature Portfolio policies, see our Editorial Policies and the Editorial Policy Checklist.

Statistics

For all statistical analyses, confirm that the following items are present in the figure legend, table legend, main text, or Methods section.

n/a	Confirmed	
☐	☒	The exact sample size (*n*) for each experimental group/condition, given as a discrete number and unit of measurement
☐	☒	A statement on whether measurements were taken from distinct samples or whether the same sample was measured repeatedly
☐	☒	The statistical test(s) used AND whether they are one- or two-sided<br>*Only common tests should be described solely by name; describe more complex techniques in the Methods section.*
☒	☐	A description of all covariates tested
☐	☒	A description of any assumptions or corrections, such as tests of normality and adjustment for multiple comparisons
☐	☒	A full description of the statistical parameters including central tendency (e.g. means) or other basic estimates (e.g. regression coefficient) AND variation (e.g. standard deviation) or associated estimates of uncertainty (e.g. confidence intervals)
☐	☒	For null hypothesis testing, the test statistic (e.g. *F*, *t*, *r*) with confidence intervals, effect sizes, degrees of freedom and *P* value noted<br>*Give P values as exact values whenever suitable.*
☒	☐	For Bayesian analysis, information on the choice of priors and Markov chain Monte Carlo settings
☒	☐	For hierarchical and complex designs, identification of the appropriate level for tests and full reporting of outcomes
☐	☒	Estimates of effect sizes (e.g. Cohen's *d*, Pearson's *r*), indicating how they were calculated

Our web collection on statistics for biologists contains articles on many of the points above.

Software and code

Policy information about availability of computer code

Data collection	MATLAB R2021a, ScanImage, WaveSurfer.
Data analysis	MATLAB, Kaleidagraph, Microsoft Excel, SnapGene, Binder, XDS, MOLREP, REFMAC, Coot, Python, CaImAn, Suite2P (github.com/MouseLand/suite2p). The code for analyzing the neuron culture screening results is available at https://github.com/ilyakolb/jGCaMP8-neuron-culture-screen. The custom code for the Spike2Fluorescence model is available at https://github.com/zqwei/Spike2Fluorescence_jGCaMP8. Example python code for usage of the in vivo mouse cell-attached dataset is available at https://github.com/rozmar/jGCaMP8_ground_truth_dataset.

For manuscripts utilizing custom algorithms or software that are central to the research but not yet described in published literature, software must be made available to editors and reviewers. We strongly encourage code deposition in a community repository (e.g. GitHub). See the Nature Portfolio guidelines for submitting code & software for further information.

Data

Policy information about availability of data

All manuscripts must include a data availability statement. This statement should provide the following information, where applicable:

- Accession codes, unique identifiers, or web links for publicly available datasets
- A description of any restrictions on data availability
- For clinical datasets or third party data, please ensure that the statement adheres to our policy

Most datasets generated for characterizing the new sensors are included in the published article (and its Supplementary Information files). In vivo mouse cell-attached datasets are available on the DANDI Archive (https://dandiarchive.org/dandiset/000168). Additional datasets are available from the corresponding authors on reasonable request.

Field-specific reporting

Please select the one below that is the best fit for your research. If you are not sure, read the appropriate sections before making your selection.

☒ Life sciences ☐ Behavioural & social sciences ☐ Ecological, evolutionary & environmental sciences

For a reference copy of the document with all sections, see nature.com/documents/nr-reporting-summary-flat.pdf

Life sciences study design

All studies must disclose on these points even when the disclosure is negative.

Sample size

For the cultured neuron assay, we used n consistent with our power analysis from an earlier study (PLoS ONE 8: e77728); prioritized variants received many more replicates (11, 24, and 64 for the three jGCaMP8 indicators). We found the purified protein experiments to be extremely reproducible, with n of 3-5 routinely providing very small error bars. Many individuals were used for each in vivo experiment, with multiple trials per field-of-view per individual - values of n ranged into the 100's for these experiments. All multiple-comparison experiments were verified to provide sufficient power with the Kruskal-Wallis multiple-comparison test before proceeding to pairwise comparison tests.

Data exclusions

No data were excluded in this study.

Replication

All replicates were successful. All group data is shown in every instance except for fluorescence images and traces, which in all cases were representative of all replicates - averaged traces with error including every point is shown in every appropriate instance. The # of replicates and/or independent experiments are noted in the figure legends and Methods; this was always at least 3 and usually more.

Randomization

There were no experimental groups, thus no need for randomization.

Blinding

There were no experimental groups, thus no need for blinding.

Reporting for specific materials, systems and methods

We require information from authors about some types of materials, experimental systems and methods used in many studies. Here, indicate whether each material, system or method listed is relevant to your study. If you are not sure if a list item applies to your research, read the appropriate section before selecting a response.

Materials & experimental systems

n/a	Involved in the study	
☐	☒	Antibodies
☐	☒	Eukaryotic cell lines
☒	☐	Palaeontology and archaeology
☐	☒	Animals and other organisms
☒	☐	Human research participants
☒	☐	Clinical data
☒	☐	Dual use research of concern

Methods

n/a	Involved in the study	
☒	☐	ChIP-seq
☒	☐	Flow cytometry
☒	☐	MRI-based neuroimaging

Antibodies

Antibodies used

1. Rabbit anti-GFP (Invitrogen, #G10362). 2. AlexaFluor 594 conjugated goat anti-Rb (Invitrogen, #A-11012). 3. chicken anti-GFP (Thermo Fisher A10262). 4. Secondary goat anti-chicken AlexaFluor 488 plus (Thermo Fisher A32931). 5. rabbit anti-RFP (Clontech 632496). 6. secondary goat anti-rabbit Cy3 (Jackson 111-165-144). 7. rat anti-GFP (AlexaFluor 488, Molecular Probes A-21311). 8. Rat anti-RFP (mAb 5F8 Chromotek). 9. secondary goat anti-rat Cy3 (Jackson 112-165-167). 10. rabbit anti-GFP (Millipore Sigma PC408). 11. Secondary goat anti-rabbit IgG conjugated to horseradish peroxidase (HRP; Thermo Fisher/Invitrogen 31460). 12. Mouse IgM anti-α-actin (Thermo Fisher/Invitrogen MA1-744). 13. Goat anti-mouse IgG and IgM-HRP (Thermo Fisher/Invitrogen 31430 and 62-6820).

Validation

All of these antibodies are routinely used in 1000's of publications a year, including essentially all from Janelia. Our only antibody labeling was against the super-common GFP & RFP epitopes, and then secondary antibodies using the most common species.
Primary chicken anti-GFP (Thermo Fisher A10262) validated in immunofluorescence here: https://www.thermofisher.com/antibody/product/GFP-Antibody-Polyclonal/A10262
Primary rabbit anti-RFP (Clontech 632496) validated extensively in immunofluorescence. Summary and publications here: https://www.takarabio.com/learning-centers/gene-function/fluorescent-proteins/fluorescent-protein-antibody-citations/rfp-antibody-citations
Primary rat anti-GFP (Molecular Probes A-21311) validated extensively in immunofluorescence. Summary and publications here: https://www.thermofisher.com/antibody/product/GFP-Antibody-Polyclonal/A-21311
Primary rat anti-RFP (mAb 5F8 Chromotek) validated extensively in immunofluorescence. Summary and publications here: https://www.ptglab.com/products/RFP-antibody-5F8.htm#publications

Primary rabbit anti-GFP (Millipore Sigma PC408) validated extensively in Westerns. Summary and publications here: https://www.emdmillipore.com/US/en/product/Anti-Green-Fluorescent-Protein-26-39-Rabbit-pAb,EMD_BIO-PC408#anchor_REF
Primary mouse IgM anti-α-actin (Thermo Fisher/Invitrogen MA1-744) validated extensively in Westerns. Summary and publications here: https://www.thermofisher.com/antibody/product/Actin-Antibody-clone-mAbGEa-Monoclonal/MA1-744
Primary rabbit anti-GFP (Invitrogen, #G10362) validated extensively in immunofluorescence. Summary and publications here: https://www.thermofisher.com/antibody/product/GFP-Antibody-Recombinant-Monoclonal/G10362

Eukaryotic cell lines

Policy information about cell lines

Cell line source(s)	No cell lines were used. Cultured neurons were derived acutely from rats and only used for short-term experiments.
Authentication	NA
Mycoplasma contamination	NA
Commonly misidentified lines (See ICLAC register)	NA

Animals and other organisms

Policy information about studies involving animals; ARRIVE guidelines recommended for reporting animal research

Laboratory animals	Neonatal (P0, both sexes) rat pups (Sprague-Dawley, Charles River Laboratory) were used for neuronal culture. Adult (female, 3-5 days after eclosure) & larval (female 3rd instar) flies of various genotypes listed in the paper were used. Young adult (postnatal day 50-214) male C57BL/6J (Jackson Labs) were used for cortex experiments. Young adult (postnatal day 42-98) male C57BL/6J (Jackson Labs) mice were used for cerebellum experiments.
Wild animals	No wild animals were used in the study.
Field-collected samples	No field collected samples were used in the study.
Ethics oversight	The HHMI Janelia Campus IACUC & IBC committees oversaw the cortex work. The Princeton University IACUC & IBC committees oversaw the cerebellum work.

Note that full information on the approval of the study protocol must also be provided in the manuscript.
